# Endoscopic features of oxyntic gland adenoma and gastric adenocarcinoma of the fundic gland type differ between patients with and without *Helicobacter pylori* infection: a retrospective observational study

**DOI:** 10.1186/s12876-022-02368-w

**Published:** 2022-06-12

**Authors:** Masaya Iwamuro, Chiaki Kusumoto, Masahiro Nakagawa, Kazuhiro Matsueda, Sayo Kobayashi, Masao Yoshioka, Tomoki Inaba, Tatsuya Toyokawa, Chihiro Sakaguchi, Shouichi Tanaka, Takehiro Tanaka, Hiroyuki Okada

**Affiliations:** 1 Department of Gastroenterology and Hepatology, Okayama University Graduate School of Medicine, Dentistry, and Pharmaceutical Sciences, 2-5-1 Shikata-cho, Kita-ku, Okayama, Okayama 700-8558 Japan; 2 Department of Gastroenterology, Nippon Kokan Fukuyama Hospital, 1844 Tsunoshita, Daimon-cho, Fukuyama, Hiroshima Japan; 3 Department of Endoscopy, Hiroshima City Hospital, 7-33 Motomachi, Naka-ku, Hiroshima, 730-8518 Japan; 4 Department of Gastroenterology and Hepatology, Kurashiki Central Hospital, 1-1-1 Miwa, Kurashiki, Okayama 710-8602 Japan; 5 Department of Internal Medicine, Fukuyama City Hospital, 5-23-1 Zao-cho, Fukuyama, Hiroshima 721-8511 Japan; 6 Department of Internal Medicine, Okayama Saiseikai General Hospital, 2-25 Kokutai-cho, Kita-ku, Okayama, Okayama 700-8511 Japan; 7 Department of Gastroenterology, Kagawa Prefectural Central Hospital, 1-2-1 Asahi-cho, Takamatsu, Kagawa 760-8557 Japan; 8 Department of Gastroenterology, Fukuyama Medical Center, 4-14-17 Okinogami-cho, Fukuyama, Hiroshima 720-8520 Japan; 9 Department of Endoscopy, National Hospital Organization Shikoku Cancer Center, 160 Kou, Minamiumemoto-cho, Matsuyama, Ehime 791-0280 Japan; 10 Department of Gastroenterology, Iwakuni Clinical Center, 1-1-1 Atago-cho, Iwakuni, Yamaguchi 740-8510 Japan; 11 Department of Pathology, Okayama University Graduate School of Medicine, Dentistry, and Pharmaceutical Sciences, 2-5-1 Shikata-cho, Kita-ku, Okayama, Okayama 700-8558 Japan

**Keywords:** Gastric neoplasms, Oxyntic gland adenoma, Gastric adenocarcinoma of the fundic gland type

## Abstract

**Background:**

The endoscopic features of oxyntic gland adenoma and gastric adenocarcinoma of the fundic gland type have not been fully investigated in relation to *Helicobacter pylori* infection status. We compared the morphology, color, and location of these lesions between patients with and without *H. pylori* infection.

**Methods:**

We retrospectively enrolled 165 patients (180 lesions) from 10 institutions. We divided the patients into the (i) Hp group (patients with current *H. pylori* infection [active gastritis, n = 13] and those with past infection [inactive gastritis, n = 76]) and (ii) uninfected group (*H. pylori*-uninfected patients, n = 52). We compared the clinical and endoscopic features of the two groups. We also performed an analysis between (i) lesions with atrophy of the surrounding gastric mucosa (atrophy group) and (ii) lesions without atrophy of the surrounding gastric mucosa (non-atrophy group).

**Results:**

The average age was older in the Hp group than in the uninfected group (68.1 ± 8.1 vs. 63.4 ± 8.7 years, *p* < 0.01). Although the difference was not statistically significant (*p* = 0.09), multiple lesions were observed in 9 of 89 patients (10.1%) in the Hp group and in only 1 of 52 patients (1.9%) in the uninfected group. Meanwhile, significant differences were observed in the prevalence of lesions located in the gastric fornix or cardia (uninfected group: 67.3% vs. Hp group: 38.0%, *p* < 0.01), with an elevated morphology (80.0% vs. 56.0%, *p* < 0.01), with a subepithelial-like appearance (78.2% vs. 42.0%, *p* < 0.01), and with a color similar to that of the peripheral mucosa (43.6% vs. 25.0%, *p* = 0.02). The male-to-female ratio, lesion size, and presence or absence of vascular dilatation or black pigmentation on the surface were not different between the two groups. In the analysis comparing lesions with and without mucosal atrophy, the prevalence of multiple lesions was significantly higher (*p* = 0.02) in the atrophy group (5/25 patients, 20.0%) than in the non-atrophy group (7/141 patients, 5.0%).

**Conclusions:**

The endoscopic features of oxyntic gland adenoma and gastric adenocarcinoma of the fundic gland type differ between patients with and without *H. pylori* infection.

**Supplementary Information:**

The online version contains supplementary material available at 10.1186/s12876-022-02368-w.

## Introduction

Oxyntic gland adenoma and gastric adenocarcinoma of the fundic gland type (GA-FG) are neoplasms composed of highly differentiated columnar cells, mainly chief cells, with pale basophilic cytoplasm and mild nuclear atypia, mimicking the oxyntic gland [1–3]. According to the World Health Organization (WHO) classification, a neoplasm confined to the mucosa is called an oxyntic gland adenoma, whereas a neoplasm with submucosal invasion is classified as GA-FG [1]. Since GA-FG was first described in a single case report in 2007 [4], this neoplasm has been predominantly observed as a subepithelial lesion in the upper third of the stomach during esophagogastroduodenoscopy [5–7]. However, some oxyntic gland adenomas and GA-FG present as flat lesions in other parts of the stomach.

We recently reported the clinicopathologic features of 126 oxyntic gland adenoma and GA-FG lesions in 116 patients from 10 institutions [8]. The lesions were classified into the following morphologic types: 0–IIa (57.1%), 0–IIb (31.0%), 0–IIc (7.1%), 0–IIa + IIc (3.2%), and 0–I (1.6%). Macroscopically, 64 lesions (50.8%) exhibited an elevated, subepithelial-like appearance. Through additional subgroup analyses, we observed that the endoscopic features of oxyntic gland adenoma and GA-FG and their predominant locations differed between patients with and without *Helicobacter pylori* infection. Therefore, in the current study, we analyzed the morphology, color, and location of these lesions in relation to *H. pylori* infection status, with more patients added to the population of our prior study.

## Methods

We retrospectively reviewed the medical charts of patients with histologically confirmed oxyntic gland adenoma and GA-FG, as previously described [8]. Histologic diagnoses were made on the basis of endoscopic biopsy, endoscopic mucosal resection, endoscopic submucosal dissection (ESD), or surgical resection. The diagnoses of oxyntic gland adenoma and GA-FG were made on the basis of the presence of intramucosal proliferation of differentiated columnar cells, which have been found to mimic the oxyntic (fundic) gland [1]. The diagnoses were supported by positive immunohistochemical staining for pepsinogen I and mucin 6 (MUC6) in some patients. We identified 165 patients who were diagnosed with oxyntic gland adenoma or GA-FG between October 2008 and November 2021. These patients were retrospectively enrolled in this study.

We retrospectively examined the *H. pylori* infection status and endoscopic features, in addition to the patients’ sex, age at diagnosis, histologic features, treatments, and prognoses. Based on the lesion morphology, depth of invasion, and histological diagnosis from the initial biopsy, the neoplastic lesions were classified according to the Japanese Classification of Gastric Carcinoma [9, 10]. Briefly, polypoid-protruding tumors were classified as type 0–I. Slightly elevated superficial tumors were defined as type 0–IIa. Superficial flat tumors without elevation or depression were classified as type 0–IIb. Slightly depressed tumors were defined as type 0–IIc. Excavated tumors with depression were classified as type 0–III. We defined an “elevated lesion” as a tumor with a 0–I, 0–IIa, or 0–IIa + IIc morphology. Biopsy results were categorized according to the group classification. For instance, Group 1 included normal tissue or non-neoplastic lesions. Group 2 included lesions that were difficult to be classified as neoplastic or non-neoplastic lesions. Group 3 included adenoma. Group 4 included neoplastic lesions suspected of being carcinomas, while Group 5 included lesions confirmed to be carcinomas [9]. The follow-up period was defined as the time from diagnosis to death of any cause or the last hospital visit. The endoscopic follow-up period was defined as the time from the endoscopic or surgical resection of the gastric lesions to the last esophagogastroduodenoscopy examination.

The *H. pylori* infection status was examined using urea breath tests, rapid urease tests, microscopic observations or culture tests of endoscopically biopsied specimens, stool antigen tests, serum or urine antibody tests, or a combination of these methods. On the basis of the results of these tests, we divided the patients into two groups according to their *H. pylori* infection status: (i) Hp group, consisting of patients with current *H. pylori* infection (active gastritis) and those with past infection (inactive gastritis), and (ii) uninfected group, consisting of *H. pylori*-uninfected patients. We also performed a secondary analysis based on the presence or absence of gastric mucosal atrophy around the lesion.

This study was approved by the ethics committees of Okayama University Hospital and the other participating institutions and was conducted in accordance with the Declaration of Helsinki. The requirement for written informed consent was waived because of the observational, non-interventional, and retrospective study design. All investigations were performed in accordance with the relevant guidelines and regulations.

## Results

The patients’ characteristics are summarized in Table 1. This study included 108 men and 57 women. The mean age at diagnosis of oxyntic gland adenoma or GA-FG was 66.3 years (range, 43–91 years). Nine patients (5.5%) had two lesions, and two patients (1.2%) had four lesions. Thus, 180 oxyntic gland adenoma and GA-FG lesions were included in this study. The lesions were removed by ESD (n = 99, 55.0%), endoscopic mucosal resection using a diathermic snare (n = 68, 37.8%), surgery (n = 3, 1.7%), or ESD followed by surgery (n = 2, 1.1%), whereas no intervention was initiated for the other eight lesions (4.4%). The resected specimens of 170 lesions were pathologically analyzed for invasion depth. The depth of invasion was T1a in 99 lesions (55.0%) and T1b in 71 lesions (39.4%). Thus, 99 oxyntic gland adenoma lesions and 71 GA-FG lesions were included in this study. During the median follow-up period of 26 months (range, 0–155 months), four patients died of causes other than oxyntic gland adenoma or GA-FG (2.4%) and two patients had no outcome information. The remaining 159 patients were alive at the last follow-up (96.4%).

**Table 1:** Clinical characteristics of the study patients

	n	%
Sex		
Male	108	65.5
Female	57	34.5
Mean age (range), years	66.3 (43–91)	
No. of lesions		
1	154	93.3
2	9	5.5
4	2	1.2
*Helicobacter pylori* infection status		
Uninfected	52	31.5
Active gastritis	13	7.9
Inactive gastritis	76	46.1
Undeterminable	24	14.5
Depth of invasion		
T1a (oxyntic gland adenoma)	99	55.0
T1b (GA-FG)	71	39.4
Not available	10	5.6
Treatments		
ESD	99	55.0
EMR	68	37.8
Surgery	3	1.7
ESD followed by surgery	2	1.1
None	8	4.4
Median follow-up period (range), months	26 (0–155)	
Median endoscopic follow-up period (range), months*	24 (0–155)	
Outcome		
Alive	159	96.4
Died of other cause	4	2.4
Unknown	2	1.2

*GA-FG* gastric adenocarcinoma of the fundic gland type, *ESD* endoscopic submucosal dissection, *EMR* endoscopic mucosal resection using a diathermic snare

*169 lesions

**Fig. 1:**
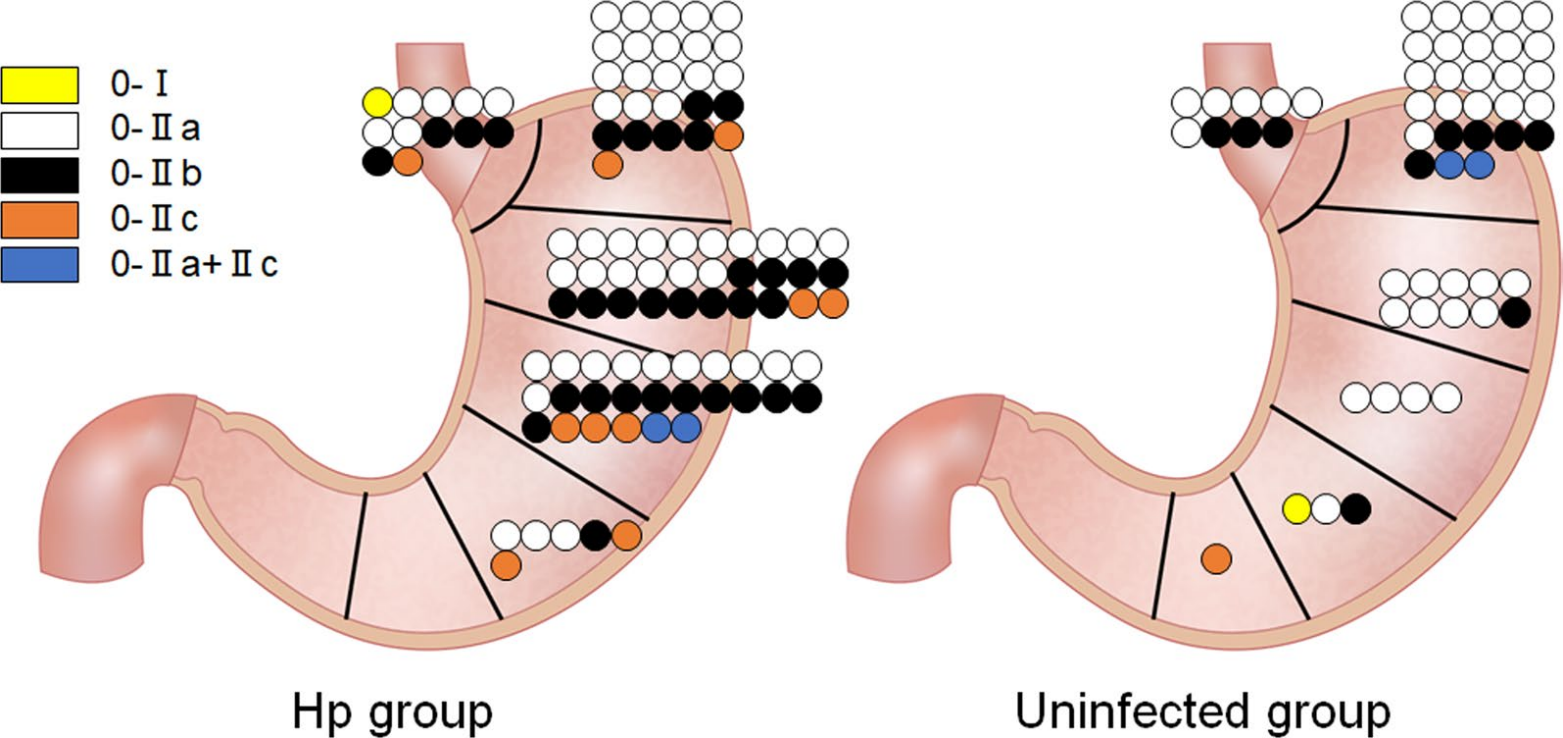
Schematic illustrations indicating the location and morphology of oxyntic gland adenoma and gastric adenocarcinoma of the fundic gland type. The stomach is divided into the fornix, cardia, body (upper, middle, and lower thirds), angle, antrum, and pylorus. Hp group, patients with current or past *Helicobacter pylori* infection

**Fig. 2:**
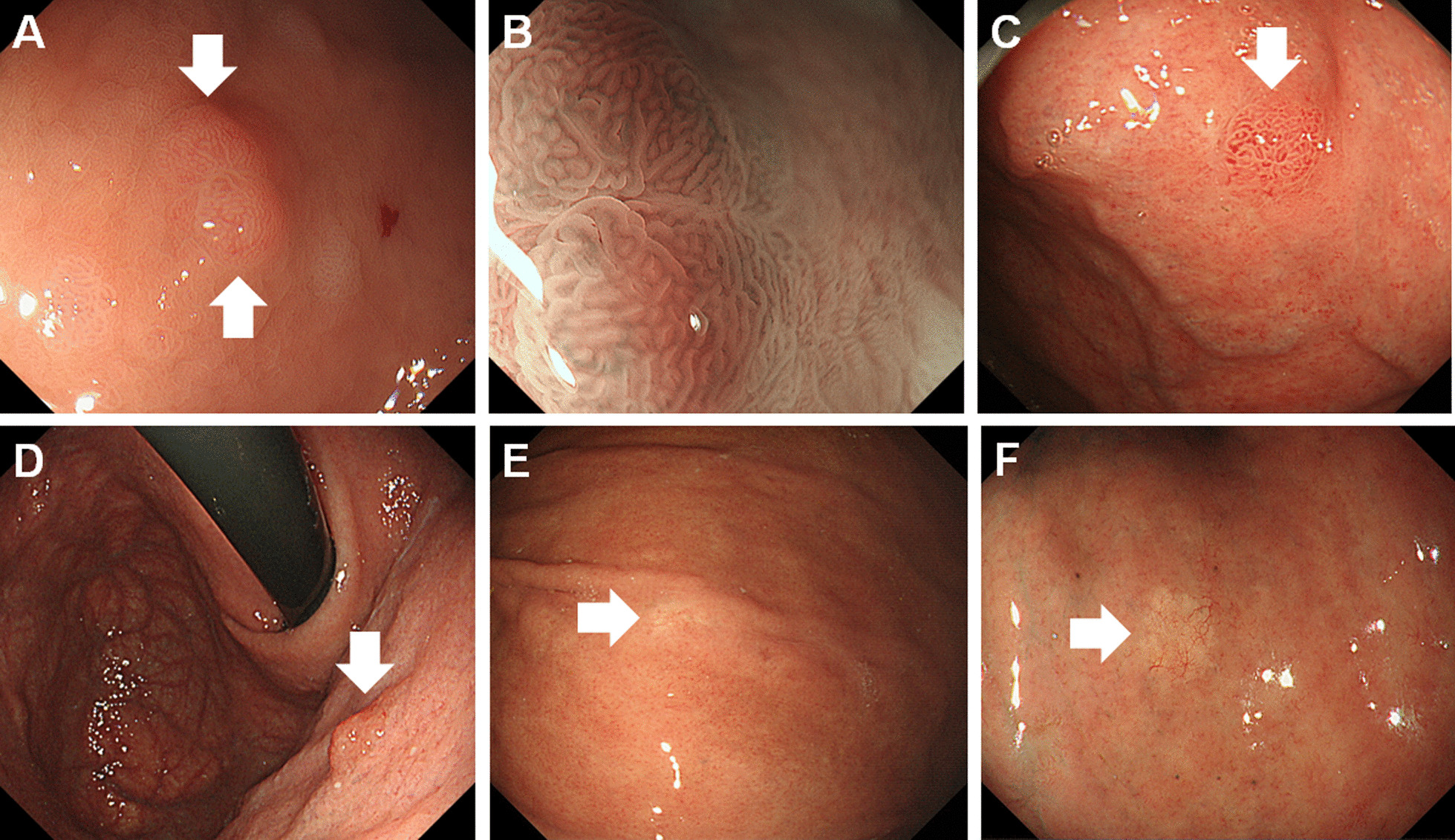
Representative images of oxyntic gland adenoma and gastric adenocarcinoma of the fundic gland type (GA-FG). **A** A 65-year-old female patient without *Helicobacter pylori* infection had an oxyntic gland adenoma with a 0-IIa, subepithelial lesion-like morphology in the gastric fornix. The color was similar to that of the surrounding gastric mucosa. **B** Magnifying endoscopy with narrow-band imaging showed dilatations of the intervening part and the crypt opening. **C** A 76-year-old female patient with current *H. pylori* infection presented with a GA-FG with deep submucosal invasion (870 μm) in the gastric fornix. The 0–IIa lesion had a reddish surface and a distinct border, resulting in a non-subepithelial lesion-like morphology. **D** A 52-year-old female patient had an oxyntic gland adenoma in the atrophic mucosa of the upper gastric body. The lesion was reddish and showed a 0–IIa, non-subepithelial lesion-like morphology. **E** A 61-year-old male patient with past *H. pylori* infection had a GA-FG that presented as a whitish, depressed (0–IIc) lesion in the middle third of the gastric body. **F** A 73-year-old male patient with past *H. pylori* infection had a GA-FG in the atrophic mucosa of the middle third of the gastric body. The lesion showed a flat (0–IIb) morphology and a yellowish–white color

With respect to *H. pylori* infection status, 52 patients (31.5%) were uninfected, 13 patients (7.9%) had active gastritis, and 76 patients (46.1%) had inactive gastritis. The *H. pylori* infection status was undeterminable in 24 patients (14.5%). Thus, 89 patients (100 lesions) were classified into the Hp group and 52 patients (55 lesions) were classified into the uninfected group. The number of test methods needed per patient to determine the *H. pylori* infection status was 1 (n = 36) or 2 (n = 16) in the uninfected group; 1 (n = 7), 2 (n = 4), 3 (n = 1), and 4 (n = 1) in the active gastritis group; 1 (n = 33), 2 (n = 14), 3 (n = 28), and 6 (n = 1) in the inactive gastritis group; and 0 (n = 23) or 1 (n = 1) in the undeterminable group. A comparison of clinical and endoscopic features between the Hp and uninfected groups is shown in Table 2. The average age of the Hp group (68.1 ± 8.1 years) was older than that of the uninfected group (63.4 ± 8.7 years) (*p* = 0.002). Although the difference was not statistically significant (*p* = 0.092), 9 of 89 patients (10.1%) in the Hp group had multiple (two or four) lesions, whereas only 1 of 52 patients (1.9%) in the uninfected group had multiple (four) lesions. Oxyntic gland adenoma or GA-FG was more frequently found in the gastric fornix or cardia in the uninfected group (37/55 lesions, 67.3%) than in the Hp group (38/100 lesions, 38.0%) (*p* < 0.001, Fig. 1). With respect to macroscopic features, 44 of 55 lesions (80.0%) in the uninfected group showed elevation (0–I, 0–IIa, or 0–IIa + IIc), whereas 56 of 100 lesions (56.0%) in the Hp group appeared as elevated lesions (*p* = 0.003). Subepithelial-like lesions were more frequently observed in the uninfected group (43/55 lesions, 78.2%) than in the Hp group (42/100 lesions, 42.0%) (*p* < 0.001). A color similar to that of the peripheral mucosa was the most common lesion color in the uninfected group (24/55 lesions, 43.6%). In contrast, whitish (30/100, 30.0%) was most predominant lesion color, followed by yellowish–white (29/100, 29.0%), in the Hp group. Therefore, the prevalence of oxyntic gland adenoma or GA-FG showing a color similar to that of the peripheral mucosa was different between the groups (43.6% vs. 25.0%, *p* = 0.017). The male-to-female ratio, lesion size, and presence or absence of vascular dilatation or black pigmentation on the surface were not different between the two groups. Representative images of oxyntic gland adenoma and GA-FG in the Hp and uninfected groups are shown in Fig. 2.

**Table 2:** Comparison of the Hp and uninfected groups

	Hp group		Uninfected group		*p* value
	n	%	n	%	
Sex					0.550
Male	59	66.3	37	71.2	
Female	30	33.7	15	28.8	
Mean age ± SD, years	68.1 ± 8.1		63.4 ± 8.7		0.002
No. of lesions					0.091*
1	80	89.9	51	98.1	
2	8	9.0	0	0.0	
4	1	1.1	1	1.9	
Mean size ± SD, mm	6.4 ± 4.5		6.6 ± 4.1		0.728
Depth of invasion					0.541^†^
T1a (oxyntic gland adenoma)	56	56.0	27	49.1	
T1b (GA-FG)	42	42.0	25	45.5	
Not available	2	2.0	3	5.5	
Location					0.001^‡^
Fornix	26	26.0	28	50.9	
Cardia	12	12.0	9	16.4	
Body	62	62.0	17	30.9	
Upper third of the body	30		10		
Middle third of the body	26		4		
Lower third of the body	6		3		
Angle	0	0.0	1	1.8	
Antrum	0	0.0	0	0.0	
Pylorus	0	0.0	0	0.0	
Morphology					0.003^§^
0–I	1	1.0	1	1.8	
0–IIa	53	53.0	41	74.5	
0–IIb	34	34.0	10	18.2	
0–IIc	10	10.0	1	1.8	
0–IIa + IIc	2	2.0	2	3.6	
0–III	0	0.0	0	0.0	
Macroscopic appearance					< 0.001
SEL-like	42	42.0	43	78.2	
Non SEL-like	58	58.0	12	21.8	
Color					0.017^‖^
Similar to the color of the peripheral mucosa	25	25.0	24	43.6	
Reddish	11	11.0	9	16.4	
Whitish	30	30.0	10	18.2	
Yellowish–white	29	29.0	9	16.4	
Yellowish	5	5.0	3	5.5	
Vascular dilatation on the surface					0.250
Present	66	66.0	34	61.8	
Absent	34	34.0	21	38.2	
Black pigmentation on the surface					0.822
Present	15	15.0	9	16.4	
Absent	85	85.0	46	83.6	

The Hp group comprised patients with current *Helicobacter pylori* infection (active gastritis) and those with past infection (inactive gastritis). The uninfected group comprised *H. pylori*-uninfected patients

*SD* standard deviation, *GA-FG* gastric adenocarcinoma of the fundic gland type, *SEL* subepithelial lesion

*Solitary versus multiple lesions

^†^T1a versus T1b

^‡^Fornix and cardia versus others

^§^Elevated (0–I, 0–IIa, or 0–IIa + IIc) versus depressed (other)

^‖^Similar to the color of the peripheral mucosa versus other

Subgroup analysis comparing the active and inactive gastritis groups revealed that the average age of patients in the active gastritis group (63.4 ± 8.2 years) was lower than that of patients in the inactive gastritis group (69.0 ± 7.9 years) (*p* = 0.037) (Additional file 1: Table S1). However, no differences were observed concerning other clinical and endoscopic characteristics, including sex, the number of lesions, mean lesion size, depth of invasion, location, morphology, macroscopic appearance, color, presence or absence of vascular dilatation, and black pigmentation on the surface.

Subsequently, we investigated the atrophy status of the gastric mucosa surrounding each oxyntic gland adenoma or GA-FG and subdivided the lesions into two groups based on the presence or absence of atrophy. The characteristics of the patients and the endoscopic features of the lesions in the two groups are presented in Tables 3 and 4. One female patient had four lesions, two of which were associated with atrophy of the surrounding mucosa and the other two were not. Thereby, the atrophy group consisted of 12 male and 13 female patients, whereas the non-atrophy group included 96 male and 45 female patients. Although the difference was not significant (*p* = 0.052), the proportion of male patients was higher in the non-atrophy group (68.1%) than in the atrophy group (48.0%). The prevalence of multiple lesions was 20.0% (5/25 patients) in the atrophy group, which was significantly higher than that in the non-atrophy group (5.0%, 7/141 patients) (*p* = 0.020). The diagnosis from the initial biopsy was non-neoplastic (Group 1 or 2) in 3/30 lesions (10.0%) in the atrophy group and 14/150 lesions (9.3%) in the non-atrophy group. The lesions in the non-atrophy group more frequently appeared in the gastric fornix or cardia (61.3% vs. 6.7%, *p* < 0.001), as elevated lesions (65.3% vs. 46.7%, *p* = 0.035), and as subepithelial-like lesions (58.0% vs. 26.7%, *p* = 0.002) than those in the atrophy group. The lesion size, invasion depth, lesion color, and presence or absence of vascular dilatation or black pigmentation on the surface were not different between the two groups.

**Table 3:** Comparison of patients with and without atrophy of the surrounding mucosa

	Atrophy group		Non-atrophy group		*p* value
	n	%	n	%	
Sex					0.052
Male	12	48.0	96	68.1	
Female	13*	52.0	45*	31.9	
Mean age ± SD, years	69.7 ± 9.2		65.7 ± 9.1		< 0.001
No. of lesions					0.020^†^
1	20	80.0	134	95.0	
2	5*	20.0	6*	4.3	
4	0	0.0	1	0.7	
Diagnosis from the initial biopsy					1.000^‡^
Group 1	0	0.0	7	4.7	
Group 2	3	10.0	7	4.7	
Group 3	1	3.3	4	2.7	
Group 4	6	20.0	18	12.0	
Group 5	20	66.7	114	76.0	

The atrophy group comprised lesions with atrophy of the surrounding gastric mucosa. The non-atrophy group comprised lesions without atrophy of the surrounding gastric mucosa

*SD* standard deviation

*One female patient had two lesions with atrophy of the peripheral mucosa and two lesions without atrophy of the peripheral mucosa

^†^Solitary versus multiple lesions

^‡^Groups 1 or 2 versus Groups 3–5

**Table 4:** Comparison of lesions with and without atrophy of the surrounding mucosa

	Atrophy group		Non-atrophy group		*p* value
Mean size ± SD, mm		7.8 ± 7.2	5.9 ± 3.6		0.176
Depth of invasion					0.963^†^
T1a (oxyntic gland adenoma)	17	56.7	82	54.7	
T1b (GA-FG)	12	40.0	59	39.3	
Not available	1	3.3	9	6.0	
Location					< 0.001^‡^
Fornix	2	6.7	66	44.0	
Cardia	0	0.0	26	17.3	
Body	28	93.3	57	38.0	
Upper third of the body	11		31		
Middle third of the body	17		16		
Lower third of the body	0		10		
Angle	0	0.0	1	0.7	
Antrum	0	0.0		0.0	
Pylorus	0	0.0		0.0	
Morphology					0.035^§^
0–I	0	0.0	3	2.0	
0–IIa	13	43.3	91	60.7	
0–IIb	13	43.3	43	28.7	
0–IIc	3	10.0	9	6.0	
0–IIa + IIc	1	3.3	4	2.7	
0–III	0	0.0	0	0.0	
Macroscopic appearance					0.002
SEL-like	8	26.7	87	58.0	
Non SEL-like	22	73.3	63	42.0	
Color					0.196^‖^
Similar to the color of the peripheral mucosa	6	20.0	51	34.0	
Reddish	3	10.0	17	11.3	
Whitish	9	30.0	38	25.3	
Yellowish–white	10	33.3	38	25.3	
Yellowish	2	6.7	6	4.0	
Vascular dilatation on the surface					0.584
Present	17	56.7	93	62.0	
Absent	13	43.3	57	38.0	
Black pigmentation on the surface					1.000
Present	4	13.3	24	16.0	
Absent	26	86.7	126	84.0	

The atrophy group comprised lesions with atrophy of the surrounding gastric mucosa. The non-atrophy group comprised lesions without atrophy of the surrounding gastric mucosa

*SD* standard deviation, *GA-FG* gastric adenocarcinoma of the fundic gland type, *SEL* subepithelial lesion

^†^T1a versus T1b

^‡^Fornix and cardia versus others

^§^Elevated versus depressed

^‖^Similar to the color of the peripheral mucosa versus other

## Discussion

We previously reported the clinicopathologic features of 116 patients with 126 oxyntic gland adenoma or GA-FG lesions and concluded that endoscopic resection is a suitable initial treatment strategy for these neoplasms [8]. In the current study, we added 49 patients with 54 oxyntic gland adenoma and GA-FG lesions to the previous study population, resulting in the largest number of lesions investigated to date.

A small, elevated lesion with a subepithelial tumor-like appearance occurring in the upper third of the stomach is a representative endoscopic feature of oxyntic gland adenoma or GA-FG [3, 4, 11–21]. Benedict et al. reviewed 111 previously reported cases and described that most tumors (89 cases, 80.2%) occurred in the upper third of the stomach [2]. Ueyama et al. reported a similar proportion of lesions in the upper third of the stomach (79/100, 79.0%) [6]. They also reported that 62 of the 100 lesions (62.0%) showed a protruded morphology. In the current study, the *H. pylori*-uninfected group showed the following typical features of oxyntic gland adenoma or GA-FG: (i) frequent occurrence in the gastric fornix or cardia; (ii) elevated, subepithelial lesion-like morphology; and (iii) a color similar to that of the peripheral mucosa. Conversely, in patients with current or past *H. pylori* infection, oxyntic gland adenoma and GA-FG appeared as flat lesions in atypical locations, rather than in the upper third of the stomach. To our knowledge, this is the first study to investigate the difference in the endoscopic features of oxyntic gland adenoma and GA-FG between patients with and without *H. pylori* infection.

*H. pylori* infection is a major causative factor in the development of conventional gastric cancer through chronic inflammation, in addition to environmental, dietary, and genetic factors [22]. In contrast, oxyntic gland adenoma and GA-FG are believed to develop independently of *H. pylori* infection, as most of these neoplasms arise in areas with no apparent atrophy endoscopically or pathologically [23]. In the present study, atrophic changes were not identified endoscopically in the surrounding mucosa in 150 of the 180 lesions (83.3%). Ueyama et al. investigated the clinicopathologic and molecular biological features of 100 lesions and found no significant differences between *H. pylori* infection-negative and *H. pylori* infection-positive or -eradicated groups, except for patient age and Ki-67 MIB-1 labeling index [6]. These results suggest that oxyntic gland adenoma and GA-FG have similar carcinogenesis and biological properties, regardless of the *H. pylori* infection status. However, we observed that the location and morphology were different between the Hp and uninfected groups. In addition, multiple tumors were more frequently found in the atrophy group than in the non-atrophy group (20.0% vs. 5.0%) in the present study.

On pathologic examination, the surface of these lesions is generally covered by normal-appearing foveolar-type epithelium [1, 2]. We speculate that oxyntic gland adenoma and GA-FG occurring in the gastric body, particularly flat lesions, are less detectable in patients without *H. pylori* infection owing to the non-atrophic, thick mucosa that covers the lesions. In contrast, during esophagogastroduodenoscopy in patients with *H. pylori* infection, these lesions may be more noticeable in the mucosa, which shows atrophic change, inflammation, or metaplasia due to the infection. Another hypothesis is that oxyntic gland adenoma and GA-FG may be misinterpreted as fundic gland polyps and may be underdiagnosed in *H. pylori*-uninfected patients. These hypotheses might explain why the location, morphology, color, and number of lesions differed between patients with and without *H. pylori* infection. However, further investigation is required to elucidate the nature of oxyntic gland adenoma and GA-FG in relation to *H. pylori* infection status.

As previously described, although an elevated, subepithelial lesion-like morphology is a representative endoscopic feature of oxyntic gland adenoma and GA-FG, tumors often present as flat lesions [23–30]. Ueyama et al. reported that 62 of 100 lesions (62.0%) showed a protruded morphology, whereas the remaining 38 lesions (38.0) presented as flat or depressed lesions [6]. In the present study, we observed that the flat or depressed type of oxyntic gland adenoma and GA-FG predominantly occurred in the gastric body in the Hp group (Fig. 1). As these lesions can show a whitish color (Fig. 2E, F), endoscopists may need to differentiate them from conventional gastric cancer and extranodal marginal zone lymphoma of mucosa-associated lymphoid tissue. Meanwhile, from the standpoint of pathologists, oxyntic gland adenoma and GA-FG may be mistaken for a neuroendocrine tumor or even non-neoplastic polyps on pathologic analysis [1]. Thus, endoscopists should be aware of the occurrence of oxyntic gland adenoma and GA-FG with a flat or depressed morphology in the gastric body of patients with current or past *H. pylori* infection. In addition, endoscopists and pathologists should have a similar index of suspicion for oxyntic gland adenoma and GA-FG to make an appropriate diagnosis.

This study had several limitations. First, the *H. pylori* infection status was classified into uninfected, active, and inactive gastritis based on a single test method in 76 patients. Thus, over- and underestimation of the infection status might have occurred because of false-positive and false-negative results. Second, researchers at various institutions subjectively investigated the presence or absence of atrophy in the surrounding mucosa using endoscopic images. Interobserver variations and differences in methodologies between the participating endoscopists may have resulted in interpretation bias during the analysis of the atrophy and non-atrophy groups. Thus, pathologic evaluation of mucosal atrophy should be incorporated in the analysis of these cases. Third, immunohistochemical studies were not performed for all patients. Although a diagnosis can essentially be made according to the WHO classification criteria [1], confirmation by positive immunohistochemical staining for both pepsinogen I and MUC6, as well as cell differentiation markers such as H+/K+-ATPase (parietal cell) and MUC5AC (foveolar epithelium), is preferable [5–7].

## Conclusions

We retrospectively investigated 165 patients with 180 oxyntic gland adenoma or GA-FG lesions. The lesions in the *H. pylori*-uninfected group predominantly showed typical features such as frequent occurrence in the gastric fornix or cardia, elevated and subepithelial lesion-like morphology, and a color similar to that of the peripheral mucosa. Endoscopists should be aware that these tumors may appear as flat lesions in atypical locations, instead of in the upper third of the stomach, in patients with current or past *H. pylori* infection. In addition, multiple tumors may exist in the atrophic mucosa. We believe that these features related to *H. pylori* infection status will facilitate the identification and prompt endoscopic diagnosis of oxyntic gland adenoma or GA-FG.

## Supplementary Information

**Additional file 1.** **Table S1.** Comparison of the active and inactive gastritis groups.

## Data Availability

The data that support the findings of this study are available from the corresponding author (MI) on reasonable request. The data are not publicly available due to their containing information that could compromise the privacy of research participants.
