# Supplementary material for: Endoscopic features of oxyntic gland adenoma and gastric adenocarcinoma of the fundic gland type differ between patients with and without Helicobacter pylori infection: a retrospective observational study
Source: BMC Gastroenterol. 2022 Jun 12;22:294. doi: 10.1186/s12876-022-02368-w (PMC9188703; doi:10.1186/s12876-022-02368-w)
Supplement: Supplementary file 1 — Additional file 1. Table S1. Comparison of the active and inactive gastritis groups. [file 12876_2022_2368_MOESM1_ESM.docx]

Suppl. Table 1. Comparison of the active and inactive gastritis groups

|  | Active gastritis | | Inactive gastritis | | p Value |
| --- | --- | --- | --- | --- | --- |
|  | n | % | n | % |  |
| Sex |  |  |  |  | 0.755 |
| Male | 8 | 61.5 | 51 | 67.1 |  |
| Female | 5 | 38.5 | 25 | 32.9 |  |
| Mean age ± SD, years | 63.4 ± 8.2 |  | 69.0 ± 7.9 |  | 0.037 |
| No. of lesions |  |  |  |  | 0.614^*^ |
| 1 | 11 | 84.6 | 69 | 90.8 |  |
| 2 | 2 | 15.4 | 6 | 7.9 |  |
| 4 | 0 | 0.0 | 1 | 1.3 |  |
| Mean size ± SD, mm | 6.3 ± 3.2 |  | 6.4 ± 4.7 |  | 0.920 |
| Depth of invasion |  |  |  |  | 0.573^†^ |
| T1a (oxyntic gland adenoma) | 10 | 66.7 | 46 | 54.1 |  |
| T1b (GA-FG) | 5 | 33.3 | 37 | 43.5 |  |
| Not available | 0 | 0.0 | 2 | 2.4 |  |
| Location |  |  |  |  | 1.000^‡^ |
| Fornix | 5 | 33.3 | 21 | 24.7 |  |
| Cardia | 1 | 6.7 | 11 | 12.9 |  |
| Body | 9 | 60.0 | 53 | 62.4 |  |
| Upper third of the body | 5 |  | 25 |  |  |
| Middle third of the body | 2 |  | 24 |  |  |
| Lower third of the body | 2 |  | 4 |  |  |
| Angle | 0 | 0.0 | 0 | 0.0 |  |
| Antrum | 0 | 0.0 | 0 | 0.0 |  |
| Pylorus | 0 | 0.0 | 0 | 0.0 |  |
| Morphology |  |  |  |  | 0.563^§^ |
| 0–I | 0 | 0.0 | 1 | 1.2 |  |
| 0–IIa | 8 | 53.3 | 45 | 52.9 |  |
| 0–IIb | 0 | 0.0 | 2 | 2.4 |  |
| 0–IIc | 4 | 26.7 | 30 | 35.3 |  |
| 0–IIa+IIc | 3 | 20.0 | 7 | 8.2 |  |
| 0–III | 0 | 0.0 | 0 | 0.0 |  |
| Macroscopic appearance |  |  |  |  | 1.000 |
| SEL-like | 6 | 40.0 | 36 | 42.4 |  |
| Non SEL-like | 9 | 60.0 | 49 | 57.6 |  |
| Color |  |  |  |  | 0.344^‖^ |
| Similar to the color of the peripheral mucosa | 2 | 13.3 | 23 | 27.1 |  |
| Reddish | 2 | 13.3 | 9 | 10.6 |  |
| Whitish | 4 | 26.7 | 26 | 30.6 |  |
| Yellowish–white | 6 | 40.0 | 23 | 27.1 |  |
| Yellowish | 1 | 6.7 | 4 | 4.7 |  |
| Vascular dilatation on the surface |  |  |  |  | 0.768 |
| Present | 11 | 73.3 | 55 | 64.7 |  |
| Absent | 4 | 26.7 | 30 | 35.3 |  |
| Black pigmentation on the surface |  |  |  |  | 0.118 |
| Present | 0 | 0.0 | 15 | 17.6 |  |
| Absent | 15 | 100.0 | 70 | 82.4 |  |

SD, standard deviation; GA-FG, gastric adenocarcinoma of the fundic gland type; SEL, subepithelial lesion.

^*^Solitary vs. multiple lesions. ^†^T1a vs. T1b. ^‡^Fornix and cardia vs. others. ^§^Elevated (0–I, 0–IIa, or 0–IIa+IIc) vs. depressed (other). ^‖^Similar to the color of the peripheral mucosa vs. other.

The active gastritis group comprised patients with current *Helicobacter pylori* infection. The inactive gastritis group comprised patients with past infection of *H. pylori*.
